# Granulocyte Transfusion Combined with Granulocyte Colony Stimulating Factor in Severe Infection Patients with Severe Aplastic Anemia: A Single Center Experience from China

**DOI:** 10.1371/journal.pone.0088148

**Published:** 2014-02-05

**Authors:** Huaquan Wang, Yuhong Wu, Rong Fu, Wen Qu, Erbao Ruan, Guojin Wang, Hong Liu, Jia Song, Limin Xing, Jing Guan, Lijuan Li, Chunyan Liu, Zonghong Shao

**Affiliations:** Department of Hematology, General Hospital, Tianjin Medical University, Tianjin, China; University of Sao Paulo - USP, Brazil

## Abstract

**Objective:**

To investigate the efficacy and safety of granulocyte transfusion combined with granulocyte colony stimulating factor (G-CSF) in severe infection patients with severe aplastic anemia (SAA).

**Methods:**

Fifty-six patients in severe infections with SAA who had received granulocyte transfusions combined with G-CSF from 2006 to 2012 in our department were analyzed. A retrospective analysis was undertaken to investigate the survival rates (at 30 days, 90 days and 180 days), the responses to treatment (at 7 days and 30 days, including microbiological, radiographic and clinical responses), the neutrophil count and adverse events after transfusion.

**Results:**

All SAA patients with severe infections were treated with granulocyte transfusions combined with G-CSF. Forty-seven patients had received antithymocyte globulin/antilymphocyte globulin and cyclosporine A as immunosuppressive therapy. The median number of granulocyte components transfused was 18 (range, 3–75). The survival at 30 days, 90 days and 180 days were 50(89%), 39(70%) and 37(66%) respectively. Among 31 patients who had invasive fungal infections, the survival at 30 days, 90 days and 180 days were 27(87%), 18(58%) and 16(52%) respectively. Among the 25 patients who had refractory severe bacterial infections, the survival at 30 days, 90 days and 180 days were 23(92%), 21(84%) and 21(84%) respectively. Survival rate was correlated with hematopoietic recovery. Responses of patients at 7 and 30 days were correlated with survival rate. Common adverse effects of granulocyte transfusion included mild to moderate fever, chills, allergy and dyspnea.

**Conclusion:**

Granulocyte transfusions combined with G-CSF could be an adjunctive therapy for treating severe infections of patients with SAA.

## Introduction

Aplastic anemia (AA) is a hematologic disease characterized by peripheral pancytopenia with bone marrow failure, and has the absence of an abnormal infiltrates and no increase in reticulin. Severe aplastic anemia (SAA) has more severe bone marrow failure and high mortality, which has been recognized an immune-mediated destruction of hematopoietic cells caused by active T lymphocytes [1]–[3]. Death of SAA is usually due to complications such as infection, hemorrhage, or severe anemia. Fatal infection by bacteria or invasive fungal (especially *Aspergillus*) is the most frequent cause of SAA mortality [4]–[8]. Even in the last few years, the response rate of new antifungal drugs, as Voriconazole and liposomal amphotericin B, was only about 30% in patients with severe neutropenia and persistent fever [9].

Neutropenia could increase the risk of infections caused by bacteria and invasive fungi. The morbidity of infection was correlated with the severity and duration of neutropenia. In recent, a random control study reported the efficacy of granulocyte colony stimulating factor (G-CSF) in severe infection of patients with very severe aplastic anemia (VSAA) [10]. And some studies indicated granulocyte transfusion increased the response rate in patients with SAA and other severe neutropenia diseases [11]–[15]. In our study, we analyzed the efficacy and safety of granulocyte transfusion combining with G-CSF in severe infections of SAA patients.

## Patients and Methods

### Subjects

Fifty-six SAA patients with infections (35 males and 21 females, median age 29 (range 6–65 years)) were enrolled in this study. A retrospective analysis was undertaken. All the patients were diagnosed in our department from 2006 to 2012. The diagnosis of SAA was defined as pancytopenia with at least two of the following abnormalities: a neutrophil count less than 0.5×10^9^/L, a platelet count less than 20×10^9^/L, and a reticulocyte count less than 20×10^9^/L with hypocellular bone marrow (less than 30% cellularity). VSAA was diagnosed in the cases SAA with the neutrophil count <0.2×10^9^/L [16]–[17]. Patients were excluded if they had congenital AA. Patients were screened for paroxysmal nocturnal hemoglobinuria (PNH) by flow cytometry using anti-CD55 and anti-CD59 antibodies. All the patients had received bone marrow cytogenetic examinations.

Among 56 patients, 51 cases were VSAA and 5 cases were SAA. Forty-seven of them had received immunosuppressive therapy (IST), including: 33 patients had received rabbit anti-human lymphocyte globulin (ALG) + cyclosporine-A (CsA), 6 patients had received rabbit anti-human thymocyte globulin (ATG) + CsA, and 8 patients had received pig ALG + CsA. The rest 9 patients had received CsA + androgen as treatments of SAA (Table 1).

**Table 1:** Characteristics of SAA patients received granulocytes and G-CSF therapy.

		Num. of patients
Gender	male	35
	female	21
Median age inyears(range)	29 (6–65)	
Severity of AA	VSAA	51
	SAA	5
Therapy	Rabbit ATG + CsA	6
	Rabbit ALG + CsA	33
	Pig ALG + CsA	8
	CsA + Androgen	9

### Characteristics of Severe Infection in Patients with SAA

Most infections in 56 patients were polymicrobial, involving more than one bacterial strain, more than one mold, or mixture infections of bacteria and fungi. Among them, 31 patients had invasive fungal infection, included that 7 cases were infected by *Aspergillus* in the lung or paranasal sinus. Other fungi included *Candida albicans* and *Candida tropicalis*. Among the 25 patients who had severe bacterial infections, 16 cases had bacteremia. *Stenotrophomonas maltophilia*, *Pseudomonas aeruginosa* and *Acinetobacter baumannii* were the common pathogens separated from blood samples of these patients. Besides blood infections, lung was the most common sites of infection (Table 2).

**Table 2:** Characteristics of infections in SAA patients received granulocytes and G-CSF therapy.

	N. of patients	blood	Lung	CNS	Sinus	liver	others
Fungal	31						
Aspergillus spp	7		4		3		
Candida albicans	12	3	5				4
Candida tropicalis	4	1	2				1
Candida krusei	1		1				
Candida glabrata	1		1				
Cryptococcus neoformans	1			1			
Unknown#	5		3	1		1	
Bacterial							
Stenotrophomonas maltophilia*	7	4	7				2
Pseudomonas aeruginosa*	6	3	4				2
Acinetobacter baumannii	3	1	1				
Enterobacter cloacae	2	1	1				
MRSA	2	2					
Klebsiella pneumoniae	2	2					
Escherichia coli	2	2					
VRE	1	1					

# Unknown invasive fungal infections were clinical diagnosed (possible).

*Some patients had several infection organs simultaneously.

MRSA: methicillin-resistant staphylococcus aureus. VRE: vancomycin-resistant enterococci.

### Therapy

During and after IST, neutropenic fever was treated initially with broad-spectrum antibiotics, followed by empiric anti-fungal therapy within 48 h if fever persisted.

Patients received granulocyte transfusions and G-CSF treatment if they had the following: (i) the neutrophil count was less than 0.2×10^9^/L and expected to last for at least 10 days, and (ii) proven or probable invasive fungal disease according to the Chinese Invasive Fungal Infection Working Group criteria [18], or a bacterial infection with higher mortality in our department, and (iii) no response to appropriate antibiotic or antifungal therapy for 24–48 h.

The study was approved by the Ethics Committee of the Tianjin Medical University. Informed written consent was obtained from all patients or their relatives in accordance with the Declaration of Helsinki.

### Granulocyte Concentrates and Transfusion

All granulocyte concentrates were ABO compatible to recipients and were collected with blood cell separator (Thermo RC3BP PLUS, US) from health donors (not submitted to granulocyte mobilization) in Tianjin Blood Center. The mean granulocyte dose in concentrates was 9.2±4.7×10^9^ cells.

All granulocyte concentrates were transfused within 4–6 h after collection. All patients received anti-anaphylaxis drugs and G-CSF (5–10 ug/kg, by hypodermic injection) before transfusions. All patients who received the granulocyte transfusion had been treated daily or on alternate days until granulocyte count returned to normal, clearance of infection, discharged, or death.

### Outcome Measures

Collected data included the survival rate (at 30 days, 90 days and 180 days, from the start to granulocyte transfusions), responses to treatment (at 7 and 30 days), and neutrophil count after granulocyte therapy. Complete blood counts were tested in all patients in 4–8 h after transfusion.

Responses to anti-infection therapy were categorized by microbiological (resolution of bacteremia), radiographic (decrease in infiltrates or nodule size) and clinical criteria. The clinical criteria included defervescence or body temperature decrease at least 1.5°C, hemodynamic stabilization, and improvement in symptoms such as dyspnea. A complete response (CR) was defined as improvement in all three criteria (microbiological, radiographic and clinical); a partial response (PR) was defined as improvement in one or two criteria; stable disease was defined as no improvement; progressive disease signified clinical deterioration [11].

### Statistical Analysis

Results were presented as means ± SD unless otherwise specified. Two tailed t-test analysis was used for two groups. Pearson’s correlation coefficient was used for the correlation analysis. Differences were considered statistically significant if p value <0.05.

## Results

### Efficacy

The survival rate at 30 days, 90 days and 180 days was 89% (50 cases), 70% (39) and 66% (37) respectively. Survival was correlated with bone marrow hematopoietic recovery. Among the 31 patients who had invasive fungal infections, survival rate at 30 days, 90 days and 180 days was 87% (27), 58% (18) and 52% (16) respectively. Among the 25 patients who had refractory severe bacterial infections, survival rate at 30 days, 90 days and 180 days was 92% (23), 84% (21) and 84% (21) respectively (Table 3).

**Table 3:** Survival of SAA patients received granulocytes and G-CSF therapy.

	N. of patients	Survival at 30d	Survival at 90d	Survival at 180d
All patients	56	50(89%)	39(70%)	37(66%)
Fungal	31	27(87%)	18(58%)	16(52%)
Bacterial	25	23(92%)	21(84%)	21(84%)

The median number of granulocyte concentrates transfusion was 18 times (range, 3–75). The mean increased granulocyte count was 0.27±0.21×10^9^/L. The median interval between the initiation of IST and the initiation of granulocyte transfusion was 14 (0 to 47) days.

After granulocyte transfusion and G-CSF treatment, the response rate (CR + PR) of SAA patients with infections at 7 days and 30 days was 52% (29) and 66%(37) respectively (Table 4). Two of the 7 patients who had *Aspergillosis* infections were cured after neutrophil count returned to normal. Other 5 cases died because hematopoietic function could not recover for a long time. Although 5 patients died, their survival times were much longer than the SAA patients in *Aspergillosis* infection without granulocyte transfusion that we studied before. We had observed that most of SAA patients in invasive *Aspergillosis* infection without granulocyte transfusions or G-CSF therapy died within the first month after diagnosis compared that most of the SAA patients with granulocyte transfusions and G-CSF therapy died in the second to third month after diagnosis if their bone morrow hematopoiesis could not recover.

**Table 4:** Response of SAA patients receiving granulocytes and G-CSF therapy.

	Response at 7 d				Response at 30 d			
	CR	PR	SD	PD	CR	PR	SD	PD
All patients	11	18	21	6	22	15	6	13
Fungal	3	8	16	4	8	10	4	9
Bacterial	8	10	5	2	14	5	2	4

CR: complete response. PR: partial response. SD: stable disease. PD: progressive disease.

### Safety

Chills and fever occurred in 8.3% of granulocyte transfusions (total 1078 transfusions). In all cases, these kinds of reactions were mild or moderate, which were successfully treated and prevented in the follow transfusions by antipyretics or corticosteroids. Dyspnea occurred in 1.9% of transfusions. Baseline oxygen saturation decreases more than 5% were seen in 6.4% of transfusions, and decreases of more than 10% in 1.9%. Allergy reaction was seen in 3.4% of transfusions. Two old patients experienced acute heart failure caused by relative quick speed of transfusion, and were cured by digoxin and furosemide. There was no other severe adverse event associated with granulocyte transfusions. (Table 5).

**Table 5:** Adverse effects of SAA patients received granulocytes and G-CSF therapy.

	Percentage of transfusion (%)
Chill and fever	8.3
Dyspnea	1.9
Allergy	3.4
Heart failure	0.2

## Discussion

Infection is a very common complication of SAA. Severe infections are fatal in SAA patients (especially treated with IST) and other severe neutropenia diseases.

There are very few specific reports about infections and their therapy in patients with AA. In studies of leukemia, neutropenia was shown to increase the risk of bacterial infections. The severity and mortality of infection were significantly correlated with the severity and duration of neutropenia. In cancer studies, it has usually identified a low-risk period in therapy, determined by neutropenia. Compared with leukemia and other neutropenia induced by cytotoxic chemotherapy, neutropenia of SAA exists much longer. As severe granulocytopenia becomes prolonged, infection will be inevitable. And we had found that the patients with neutrophil count less than 0.2×10^9^/L (as VSAA patients) had more frequent and severe infection: Pulmonary infection and septicemia had higher mortality (56% and 73.3% respectively).

Some studies showed that G-CSF therapy reduced the mortality of infections in patients with VSAA. Among 12 VSAA patients treated with G-CSF, 8 patients had good response. While 13 VSAA patients without G-CSF, only 3 patients had responses to therapy [19]. The randomized controlled study by the SAA Working Party of the European Group for Blood and Marrow Transplantation showed that patients treated with G-CSF had fewer infectious episodes (24%) and hospitalization days (82%) compared with patients without G-CSF (36%; P<0.006; 87%; P<0.0003) [10]. Our past study and others studies had shown that G-CSF played an adjunctive role in severe infections in patients with VSAA [20]–[23].

Quillen et al [11] analyzed granulocyte transfusions in SAA patients with severe infections in National Institutes of Health in the past 11 years. They found granulocyte transfusions might help to increase the survival rate. The overall survival rate at hospital discharge was 58% and survival was strongly correlated with hematopoietic recovery. Among the 18 patients who had invasive fungal infections, 44% of them survived till hospital discharge. In other studies, it also showed that the administration of granulocyte transfusions to treat neutropenic patients in life-threatening infections could increase the response rate of anti-infection therapy and decrease the mortality [7]–[11].

In this study, we proved that life-threatening infections were not unusual in SAA patients, and the severity and duration of neutropenia were significantly related to the mortality of infections. We observed that granulocyte transfusions combining with G-CSF to treat severe infections in SAA patients had better responses. The survival rate at 30 days, 90 days and 180 days were 89%, 70% and 66%, which were longer than that before.

We observed that granulocyte transfusions with G-CSF could increase the response rate of antifungal and antibiotics therapy. Two patients infected by *Aspergillus* were cured successfully with Voriconizole, Caspofungin and granulocyte transfusions with G-CSF, and their hematopoiesis recovered later. In our past study, the SAA patients with *Aspergillosis* infection all died. The total mortality of SAA patients with invasive fungal infection was 61.1% and only 16.7% patients had response to antifungal therapy. All patients diagnosed invasive *Aspergillosis* died even though antifungal therapy was given suitably [24]. But after granulocyte transfusions, the neutrophil count did not increased obviously in peripheral blood. We inferred that it was due to the migration of neutrophils from blood to tissues.

According to past reports, the granulocyte concentrates collected from normal donors who stimulated by G-CSF and dexamethasone to increase neutrophil counts in most clinical centers. But in our study, granulocyte concentrates had relatively lower neutrophil counts, which collected from normal donors without stimulation. As compensation, we used G-CSF to enhance the function of neutrophils by improving the ability of migration and phagocytosis.

Though many researches showed that granulocyte transfusions and G-CSF decreased the mortality of infections, the clearance of infections still depended on the hematopoiesis recovery. Thirteen patients received neutrophil transfusions after IST survived until bone marrow function recovered. In addition, the new antifungal drugs showed more efficient to treat invasive fungal infections in this study. Hence, it would not summarize that the administration of granulocyte transfusions with G-CSF alone can treat the severe infection in SAA patients.

Adverse effects of granulocyte transfusions included mild to moderate fever, chills, allergy and dyspnea. No patient died because of granulocyte transfusions. Two old patients experienced acute heart failure because the speed of granulocyte transfusion was quick relatively, and were cured by digitalis and diuretics. There was no other severe adverse event associated with granulocyte transfusions. But we did not study the alloimmunization of granulocyte transfusion which would be investigated in further study.

In conclusion, granulocyte transfusion combining with G-CSF might be an adjunctive therapy for treating severe infections in SAA patients.
